# The *ERCC6* Gene and Age-Related Macular Degeneration

**DOI:** 10.1371/journal.pone.0013786

**Published:** 2010-11-01

**Authors:** Dominique C. Baas, Dominiek D. Despriet, Theo G. M. F. Gorgels, Julie Bergeron-Sawitzke, André G. Uitterlinden, Albert Hofman, Cornelia M. van Duijn, Joanna E. Merriam, R. Theodore Smith, Gaetano R. Barile, Jacoline B. ten Brink, Johannes R. Vingerling, Caroline C. W. Klaver, Rando Allikmets, Michael Dean, Arthur A. B. Bergen

**Affiliations:** 1 Department of Clinical and Molecular Ophthalmogenetics, The Netherlands Institute for Neuroscience (NIN), Royal Netherlands Academy of Arts and Sciences (KNAW), Amsterdam, The Netherlands; 2 Department of Epidemiology, Erasmus Medical Center, Rotterdam, The Netherlands; 3 Ophthalmology, Erasmus Medical Center, Rotterdam, The Netherlands; 4 Basic Science Program, Human Genetics Section, SAIC-Frederick, Frederick, Maryland, United States of America; 5 Department of Internal Medicine, Erasmus Medical Center, Rotterdam, The Netherlands; 6 Department of Ophthalmology, and Department of Pathology and Cell Biology, Columbia University, New York, New York, United States of America; 7 Laboratory of Experimental Immunology, Cancer and Inflammation Program, National Cancer Institute, Frederick, Maryland, United States of America; 8 Department of Ophthalmology, Academic Medical Center, University of Amsterdam, Amsterdam, The Netherlands; 9 Department of Clinical Genetics, Academic Medical Center, University of Amsterdam, Amsterdam, The Netherlands; Johns Hopkins School of Medicine, United States of America

## Abstract

**Background:**

Age-related macular degeneration (AMD) is the leading cause of irreversible visual loss in the developed countries and is caused by both environmental and genetic factors. A recent study (Tuo et al., PNAS) reported an association between AMD and a single nucleotide polymorphism (SNP) (rs3793784) in the *ERCC6* (NM_000124) gene. The risk allele also increased *ERCC6* expression. *ERCC6* is involved in DNA repair and mutations in *ERCC6* cause Cockayne syndrome (CS). Amongst others, photosensitivity and pigmentary retinopathy are hallmarks of CS.

**Methodology/Principal Findings:**

Separate and combined data from three large AMD case-control studies and a prospective population-based study (The Rotterdam Study) were used to analyse the genetic association between *ERCC6* and AMD (2682 AMD cases and 3152 controls). We also measured *ERCC6* mRNA levels in retinal pigment epithelium (RPE) cells of healthy and early AMD affected human donor eyes. Rs3793784 conferred a small increase in risk for late AMD in the Dutch population (The Rotterdam and AMRO-NL study), but this was not replicated in two non-European studies (AREDS, Columbia University). In addition, the AMRO-NL study revealed no significant association for 9 other variants spanning *ERCC6*. Finally, we determined that *ERCC6* expression in the human RPE did not depend on rs3793784 genotype, but, interestingly, on AMD status: Early AMD-affected donor eyes had a 50% lower *ERCC6* expression than healthy donor eyes (*P* = 0.018).

**Conclusions/Significance:**

Our meta-analysis of four Caucasian cohorts does not replicate the reported association between SNPs in *ERCC6* and AMD. Nevertheless, our findings on *ERCC6* expression in the RPE suggest that *ERCC6* may be functionally involved in AMD. Combining our data with those of the literature, we hypothesize that the AMD-related reduced transcriptional activity of *ERCC6* may be caused by diverse, small and heterogeneous genetic and/or environmental determinants.

## Introduction

Age-related macular degeneration (AMD) is the most common cause of irreversible blindness in the Western world. The prevalence of AMD rises sharply with age, affecting 4% of the population over the age of 60 and more than 10% of individuals older than 75 [1], [2]. The early stages of AMD are characterized by drusen, which are focal depositions of waste material underneath the retinal pigment epithelium (RPE). These early stages frequently progress over time into late AMD, which presents itself in two forms: geographic atrophy (dry AMD) and neovascular AMD (wet AMD).

As a complex disease, AMD has environmental as well as genetic determinants.

Environmental risk factors include age, smoking, hypertension and diet. Estimates of the genetic hereditability range up to 71% [3], [4]. The strongest genetic associations with AMD were found with variants in the complement factor H (*CFH*) gene [5]–[8] and with SNPs in a chromosomal region (10q26) containing the *HTRA1* and *ARMS2* genes [9], [10]. *CFH* is the main inhibitor of the alternative pathway of the complement system, and thus plays an important role in the control of the innate immune system. Recently, associations with AMD were also found for other genes of the complement cascade (*complement factor B, C2, C3* [11]–[13], but, interestingly enough, not for *C5*, although it should be noted that only relatively common SNP's in *C5* were screened [14]. Nonetheless, the accumulated evidence in the literature suggests a key role of the alternative complement pathway in AMD. Whether genetic variants in the *HTRA1* or *ARMS2* genes on 10q26, or both, are truly involved in AMD remains to be elucidated [15], [16]. In terms of function, both genes could play a role in AMD: *HTRA1* is a serine protease [9] which may affect the turnover of the ECM (i.e. Bruch's membrane), while *ARMS2* has been implicated in mitochondrial function and oxidative stress [17].

Indeed, the retina, and especially the RPE is exposed to high levels of oxidative stress [18]. The combination of high oxygen consumption, intense light exposure and the presence of photosensitizers such as lipofuscin, may lead locally to excessive oxidative damage, for example of the DNA. In this respect, the recent *ERCC6*/AMD manuscript of Tuo and coworkers (2006) is of interest. They reported that a single nucleotide polymorphism (SNP; rs3793784), in the promoter region of the *ERCC6* DNA repair gene was associated with AMD. The SNP was associated with AMD susceptibility, both independently and through interaction with a SNP (rs380390) in *CFH*.

In addition, they found that the *ERCC6* SNP altered the expression level of the gene [19].

The *ERCC6* gene is involved in DNA repair, and loss of function mutations in this gene cause Cockayne syndrome (CS) [20], [21]. CS is an autosomal recessive progeroid disorder characterized by severely impaired physical and intellectual development. Among the many clinical features, photosensitivity and pigmentary retinopathy are hallmarks of CS [21]. Cells from CS patients are specifically defective in repair of DNA lesions that are located in actively transcribed DNA and obstruct transcription [22], [23]. Substrates for this transcription-coupled repair include UV or oxidative stress induced DNA lesions [24]–[26]. DNA repair deficiency of UV-induced DNA lesions may explain photosensitivity in CS. DNA repair deficiency of oxidative stress induced DNA lesions may be implicated in other CS symptoms, including the retinopathy, and premature aging [27], [28]. Targeted disruption of *Ercc6* in the mouse resulted in a mouse model for CS [29], [30], which showed spontaneous retinal degeneration characterized by a gradual photoreceptor loss [29]. Interestingly, the retina of the *Ercc6* -/- mouse is hypersensitive to X rays, which confirmed that oxidative DNA damage is involved in CS retinal pathology.

In order to further explore the role of oxidative DNA damage in AMD, the potential association of *ERCC6* promoter SNP rs3793784: C>G *(c.-6530C>G)* with AMD was analyzed in the population-based Rotterdam study and, in parallel, in the Dutch AMRO-NL case-control study. Replication was performed in two large non-European AMD case-control (Columbia University and Age-Related Eye Disease Study (AREDS)) studies. We subsequently investigated the potential modifying effect of *CFH Y402H*, *LOC387715 A69S* and smoking. Next, nine other *ERCC6* (tag) SNPs were genotyped in 375 cases and 199 controls from the AMRO-NL study population. Finally, to examine a possible functional relationship, we determined both rs3793784 dependent and normal *ERCC6* mRNA expression levels in healthy and early AMD affected human RPE isolated from human donor eyes.

## Results

### Analysis of association for *ERCC6* promoter polymorphism rs3793784

The potential association of *ERCC6 c.-6530C>G* (rs3793784) with AMD was analyzed in the population-based Rotterdam Study and, in parallel, in the Dutch AMRO-NL case-control study. In the Rotterdam Study, 427 of 6418 persons were diagnosed with early AMD and 78 persons with late AMD at entry of the study. The mean follow-up time was 7.85 years. In this period, 1039 persons remained without AMD (“controls”), while 509 progressed to early AMD and 93 to late AMD (%). In the AMRO-NL study, 331 unrelated AMD patients and 170 controls were genotyped for rs3793784. Of those, 84 persons were diagnosed with early AMD and 247 persons with late AMD at entry of the study. Genotype frequencies were in Hardy-Weinberg Equilibrium in both studies. The population frequency of *ERCC6* G allele was 0.43. Baseline characteristics stratified for the *ERCC6 c.-6530C>G* genotype are presented in **Table S1**.

The risk of AMD for the *ERCC6 c.6530C>G* allele is summarized in **Table S2**. In the Rotterdam Study, we found a significant association between *ERCC6* and late AMD in the incident analyses. Although not statistically significant, a similar trend was seen in the prevalent analyses. In contrast, analysis of the AMRO-NL study showed no significantly increased disease OR for persons carrying potential *ERCC6* risk alleles. To determine the risk for the general Dutch population, we combined data from the Rotterdam Study and the AMRO-NL study population. This yielded a borderline significant increased risk of late AMD for persons homozygous for rs3793784 (OR 1.39, 95%CI 1.02–1.89). Analyzing subtypes of late AMD separately (dry, wet or mixed) did not yield statistical significant results: **Table S2**. We also analyzed the interaction of *ERCC6 c.-6530C>G* with three known prominent AMD risk factors in the largest combined population study available to us (The Dutch Rotterdam and AMRO-NL studies). As assessed by calculation of the synergy index (SI) no significant interaction between the *ERCC6* variant and smoking was found (SI 3.33, 95%CI 0.39–28.66). Neither did we find a significant SI for the interaction with *CFH* Y402H (SI 1.56, 95%CI 0.77–3.15) and *LOC387715* A69S (SI 3.08, 95%CI 0.83–11.50). These results implied that these risk factors did not modify the relation of *ERCC6* with AMD.

Finally, rs3793784 was also screened in two non-European study populations (Columbia and AREDS). The results from the genotype analysis of these populations are shown in **Table 1**. Again, all the genotype frequencies followed HWE (data not shown). However, in both populations, no significant associations between the SNP and AMD were found. **Table 2** shows that combining all data from the four studies did not result in statistically significant association.

**Table 1:** Risk of Age-Related Macular Degeneration for *ERCC6* c.-6530C>G Genotypes in Two Non-European Study Populations.

	No AMD (controls)	All AMD cases		Early AMD		NMD		GA		Mixed AMD		Late AMD	
**AREDS**													
***rs3793784***	N = 217	N = 921		N = 253		N = 324		N = 166		N = 178		N = 668	
	No. (%)	No. (%)	OR (95% CI)	No. (%)	OR (95% CI)	No. (%)	OR (95% CI)	No. (%)	OR (95% CI)	No. (%)	OR (95% CI)	No. (%)	OR (95% CI)
Genotype													
Noncarrier (AA)	82 (37.8)	310 (33.6)	1	83 (32.8)	1	108 (33.3)	1	56 (33.7)	1	63 (35.4)	1	227 (34.0)	1
Heterozygous (Aa)	98 (45.2)	462 (50.2)	1.25 (0.89−1.73)	136 (53.8)	1.37 (0.92−2.05)	161 (49.7)	1.25 (0.85−1.83)	80 (48.2)	1.19 (0.76−1.88)	85 (47.8)	1.13 (0.73−1.75)	326 (48.8)	1.20 (0.86−1.69)
Homozygous (aa)	37 (17.1)	149 (16.2)	1.07 (0.69−1.65)	34 (13.4)	0.91 (0.52−1.58)	55 (17.0)	1.13 (0.68−1.87)	30 (18.1)	1.19 (0.66−2.14)	30 (16.9)	1.05 (0.59−1.89)	115 (17.2)	1.13 (0.72−1.76)
MAF(%)	0.40	0.41		0.40		0.42		0.42		0.41		0.42	
**Columbia**													
***rs3793784***	N = 359	N = 365				N = 273		N = 92				N = 365	
	No. (%)	No. (%)	OR (95% CI)			No. (%)	OR (95% CI)	No. (%)	OR (95% CI)			No. (%)	OR (95% CI)
Genotype													
Noncarrier (AA)	135 (37.6)	149 (40.8)	1			115 (42.1)	1	34 (37.0)	1			149 (40.8)	1
Heterozygous (Aa)	172 (47.9)	169 (46.3)	0.89 (0.65−1.22)			124 (45.4)	0.85 (0.60−1.19)	45 (48.9)	1.04 (0.63−1.71)			169 (46.3)	0.89 (0.65−1.22)
Homozygous (aa)	52 (14.5)	47 (12.9)	0.82 (0.52−1.29)			34 (12.5)	0.77 (0.47−1.36)	13 (14.1)	0.99 (0.49−2.03)			47 (12.9)	0.82 (0.52−1.29)
MAF(%)	0.38	0.36				0.35		0.39				0.36	

AMD  =  age-related macular degeneration; MAF  =  minor allele frequency. “A” indicates common allele, “a” minor allele. Percentages not always 100% because of rounding. ORs are estimated with logistic regression analysis (with the control group as reference group and respectively early and late AMD as outcome variable).

**Table 2:** Risk of Age-Related Macular Degeneration for *ERCC6* c.-6530C>G Genotypes in all Study Populations Combined (Rotterdam, Amsterdam, Columbia University and AREDS).

	No AMD (controls)	AMD cases	
***rs3793784***	N = 3143	N = 2679	
	No. (%)	No. (%)	OR (95% CI)
Genotype			
Noncarrier (AA)	1057 (33.6)	898 (33.5)	1
Heterozygous (Aa)	1524 (48.5)	1316 (49.1)	0.89 (0.65−1.22)
Homozygous (aa)	562 (17.9)	465 (17.4)	0.82 (0.52−1.29)
MAF (%)	0.42	0.42	

AMD  =  age-related macular degeneration; MAF  =  minor allele frequency. “A” indicates common allele, “a” minor allele. Percentages not always 100% because of rounding. ORs are estimated with logistic regression analysis (with the control group as reference group and respectively early and late AMD as outcome variable).

### AMD association analysis of nine other *ERCC6* variants

**Figure 1:**
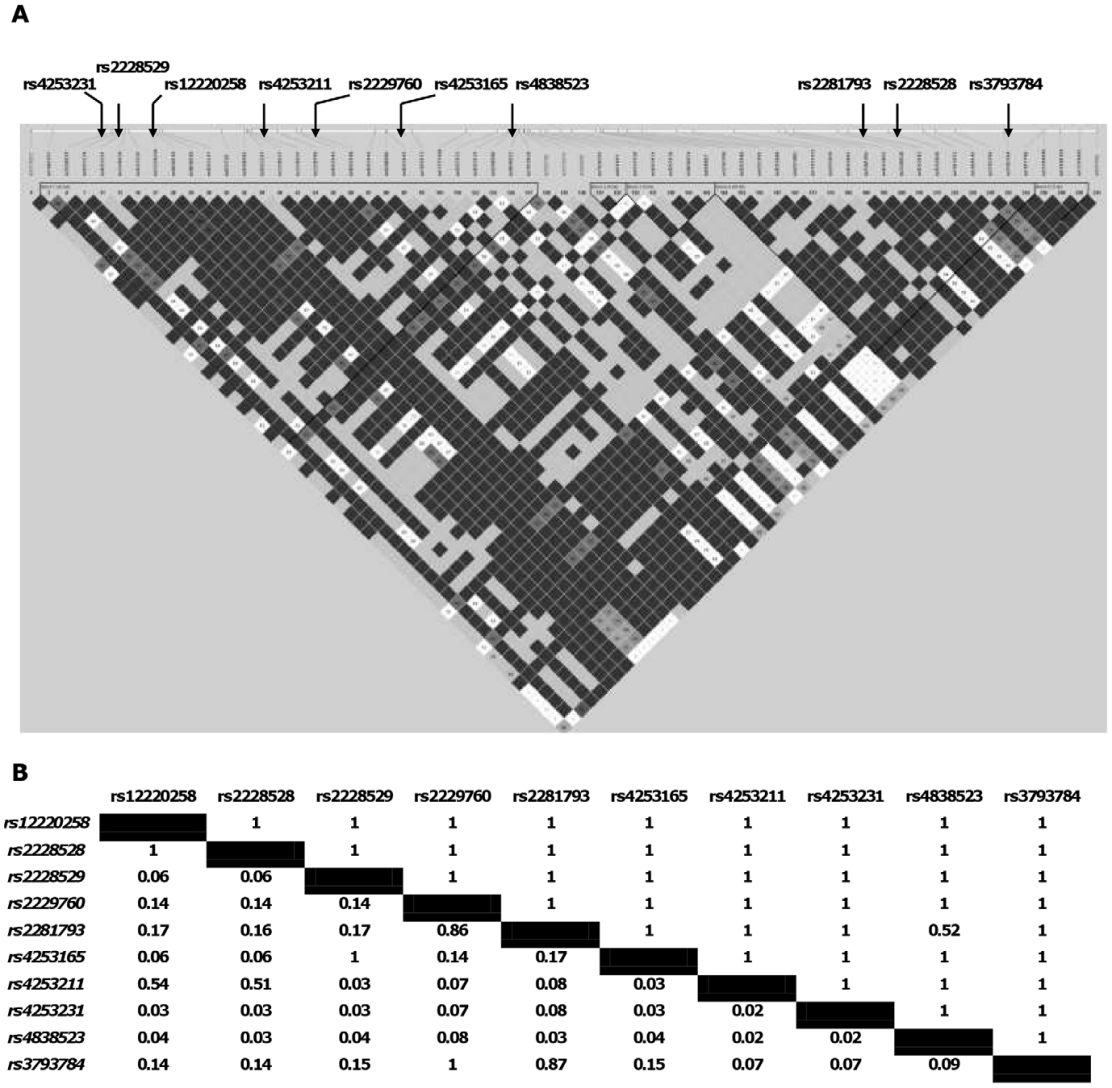
Linkage disequilibrium (LD) display in Haploview of SNPs encompassing the *ERCC6* gene. SNP selection was based on criteria like functional relevance, minor allele frequency (MAF)>10%, coverage of the main linkage disequilibrium (LD) blocks and tagging of the most common haplotypes. Tag SNPs were selected by use of Tagger, an option of Haploview [1] (all SNPs were captured with a LD tagging criteria of *r^2^*>0.8). Figure 1 displays the 5 distinct haplotype blocks and all SNPs that were tested in the AMRO-NL study population (**A**). LD scores (D' and R^2^) between markers genotyped. Note D' above the diagonal and R^2^ scores below the diagonal (**B**).

We next selected nine other *ERCC6* SNPs that span and tag the entire *ERCC6* gene. The LD plot and the distinct haplotype blocks for the nine selected SNPs, as generated by Haploview [31], are presented in **Figure 1a**. The nine variants spanned two different haplotype blocks of the *ERCC6* gene. Corresponding LD scores (D' and R^2^) for each selected marker of the *ERCC6* gene with a MAF>10% are also given (**Figure 1b**). The potential association of the SNPs with early and late AMD was analyzed in the AMRO-NL cohort. Genotype frequencies for all SNPs followed HWE (data not shown). None of the selected SNPs, including one in full LD with rs3793784 (rs2229760), showed a significant association with early or late AMD (**Table S3**).

### *ERCC6* mRNA expression in the human RPE

**Figure 2:**
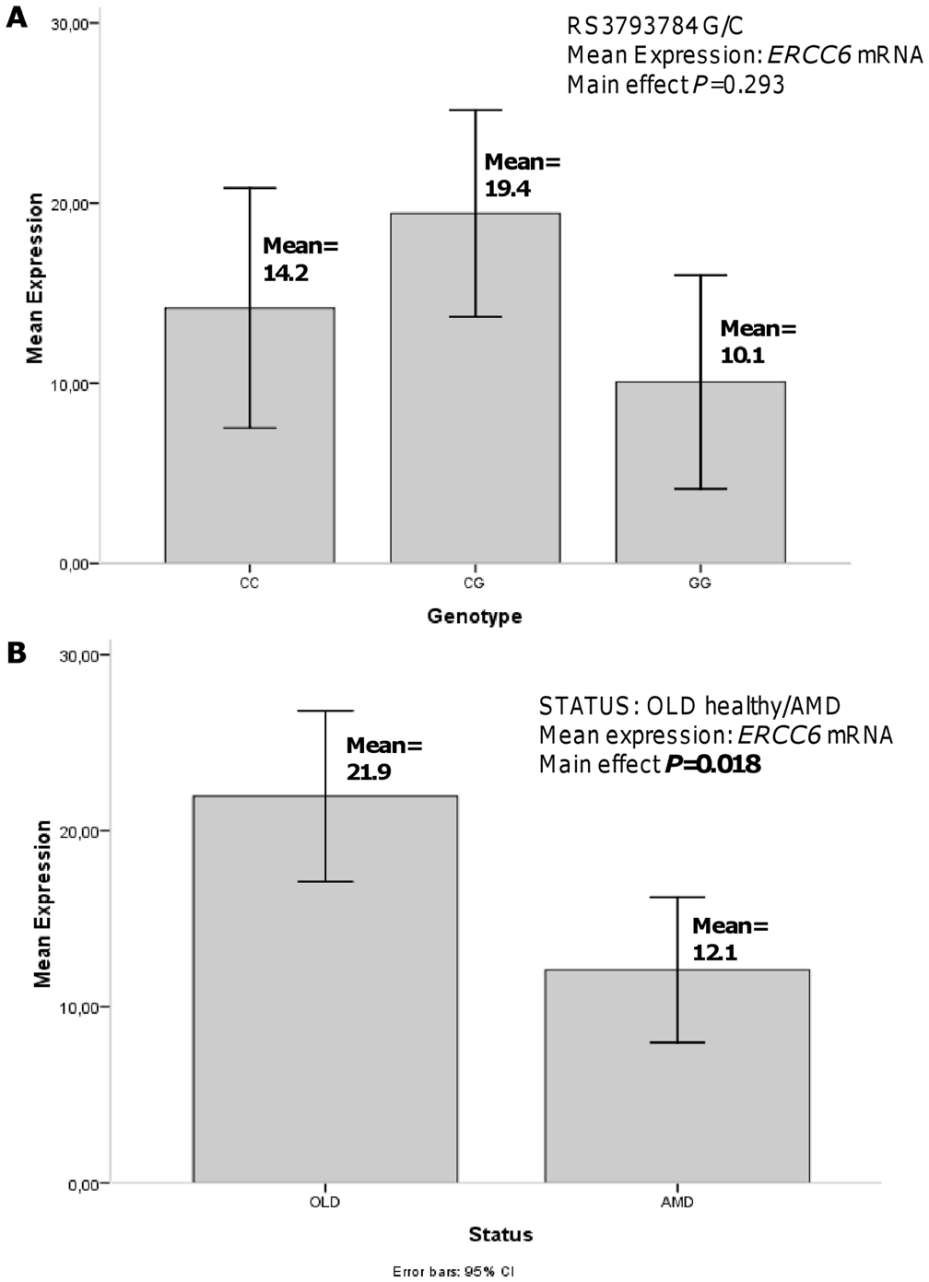
*ERCC6* expression levels in relation to genotype and disease status. Mean *ERCC6* expression level in human donor eyes in relation to rs3793784 genotype (C/C, *N* = 8; C/G, *N* = 11and G/G, *N* = 4). (**A**). Mean *ERCC6* expression level in human donor eyes in relation to “status” =  (old) healthy or early AMD (*N*  = 14 donor eyes with early AMD and 9 old healthy donor eyes) (**B**). Quantitative RT-PCR analysis, normalized to the geometric mean of three housekeeping genes (*RPLP0*, *PPIA*, and *EEF1a1*) [46], [47]. Two way ANOVA was used to test the independent effect of status and genotype on the mean expression, as well as the interaction between these variables. Abbreviations: AMD =  age-related macular degeneration.

SNP rs3793784 reportedly influences *ERCC6* gene expression, with the risk allele (G) showing higher expression [19]. We first genotyped this *ERCC6* SNP in DNA derived from the retina of 23 well characterized healthy old and early AMD affected donor eyes. We next determined *ERCC6* mRNA expression in laser dissected RPE cells from the same eyes, since this cell type occupies a central position in AMD pathology. Late AMD was not considered for RPE expression studies because of the lack of suitable cell material. There were no significant differences in age distribution between each genotype group. The results are presented in **Figures 2a and 2b**. Clearly, in the RPE, the risk allele (G) of rs3793784 did not lead to higher *ERCC6* expression levels. A two way ANOVA was used to statistically test the effect of genotype and AMD status on *ERCC6* expression. The ANOVA did not detect an independent significant effect for rs3793784 genotype on *ERCC6* expression (**Figure 2a**; *P* = 0.293). In addition, the combined effect of genotype and AMD status on ERCC6 expression was not significant either (*P_interaction_* = 0.531). However**,** the ANOVA *did* demonstrate a significant effect of AMD status on *ERCC6* expression, *independent* of the rs3793784 genotype (**Figure 2b** **,** *P* = 0.018). Interestingly, the expression of *ERCC6* in healthy RPE was nearly twice as high as in early AMD affected RPE (**Figure 2b**). The “raw” data of the three housekeeping genes and the *ERCC6* gene is presented in **Table S4**.

## Discussion

We genotyped the *ERCC6* promoter SNP rs3793784, which was previously associated with AMD, in the prospective, population based Rotterdam Study and, in parallel, in the Dutch AMRO-NL case control study. In addition, we screened two other large, non-European, study populations (Columbia University, NY and AREDS) of European-American descent. In total, this study consisted of 2682 AMD cases and 3152 ethnically matched controls.

### Non-replication of the rs3793784 SNP association with AMD

Contrary to the findings of the initial association study by Tuo et al. (2006) [19], we failed to show a (consistent) statistically significant association between *rs3793784* and AMD in all study populations.

We only detected a significant risk in the incident analysis of the Rotterdam Study, but not in the prevalent analysis. This prompted us to specifically analyze whether selective mortality may be involved: persons with two copies of *ERCC6* rs3793784 have a higher risk of AMD, and if these persons also die earlier, it could explain the discrepancy between prevalent and incident analyses. However, in the Rotterdam Study, the *ERCC6* variant was not associated with increased mortality (not shown); in addition, there was no unequal age-related genotype distribution (not shown). Thus, the small difference between the incident and prevalent risks in the population based cohort probably occurred by chance due to relatively small patient sample sizes. In the AMRO-NL case-control study, association between the rs3793784 SNP and AMD was not found. The combined data of the two Dutch populations (the Rotterdam Study and AMRO-NL study) still showed a marginally increased risk of homozygous *ERCC6* carriers for late AMD. Since this effect was largely caused by one sub-population only (incident cases and controls from the Rotterdam study), we chose to include two additional study populations. Replication of the association in two non-European study populations clearly failed: In both the Columbia University and AREDS data, no association was present between rs3793784 and AMD.

Our negative association findings in the AREDS population are of particular interest: Tuo et al. [19] previously genotyped the *ERCC6* SNP rs3793784 essentially in the *same* AREDS cohort and did find a statistically significant association. How can this discrepancy be explained? One possible explanation is the large difference in the number of AREDS participants screened: Tuo et al. [19] genotyped 460 cases and 269 controls, whereas in our AREDS screen, 929 cases and 217 controls were genotyped. Further analysis showed that the MAF of rs3793784 in the cases are comparable in both studies (0.42/0.41). However, the MAF of rs3793784 in the controls of both studies is remarkably different (AREDS Tuo: 0.35 and our AREDS study: 0.40). The Rotterdam Study, AMRO-NL study and the Columbia study yield MAFs of, respectively, 0.43, 0.42 and 0.38 in controls. Thus, we conclude that the findings of Tuo and coworkers (MAF 0.35 in the controls), and the underlying association with AMD, are most likely due to chance. In addition, in contrast with the data of Tuo et al. [19], we also did not find evidence for effect modification or interaction between *CFH* Y402H or any of the other two major AMD risk factors: smoking or the *LOC387715* A69S SNP. Overall, the possible weak association in a single Dutch sub-population and the negative association in three large replication cohorts, imply that the *ERCC6 C-6530>G* polymorphism has a minor role, if any, in the pathogenesis of AMD. This conclusion is supported by a recent study by Chen and coworkers, who conducted a genome-wide association scan for AMD in 2157 cases and 1150 controls. Amongst other, they evaluated evidence for involvement of previously associated SNPs and found no association between the *ERCC6* SNP rs3793784 and AMD [32].

Finally, it is of course possible that another genetic variant or a variant in linkage disequilibrium (LD) with *ERCC6 -6530C>G*, is a contributor to the potential AMD risk observed. In order to further elucidate the putative role of this gene in AMD we tested nine other common *ERCC6* variants in the AMRO-NL study. One of these SNPs was in perfect LD with the previously associated SNP, rs2229760. None of the selected SNPs showed a significant association with early or late AMD. These results strengthen the conclusion described above and suggest that common SNPs in the *ERCC6* gene confer no or little risk for AMD.

### *ERCC6* expression in the human RPE is related to AMD status, not to rs3793784 genotype

Tuo et al. (2006) found that rs3793784, located in the untranslated 5′ flanking region of the gene, influenced *ERCC6* expression level: the risk allele (G) resulted in 2–3 times higher *ERCC6* expression than the C variant [19]. This is an intriguing finding since it suggests that higher expression of this DNA repair gene makes the retina more susceptible to AMD. We decided to examine *ERCC6* mRNA expression in the human RPE, which occupies a central position in AMD pathology. In this cell type, we could not reproduce the paradoxical finding of Tuo and co-workers [19]. Instead, we found that *ERCC6* RPE expression levels were significantly *reduced* in early AMD affected eyes, *independent* of rs3793784 genotype.

The significantly reduced expression of *ERCC6* in the RPE of early AMD affected eyes, suggests that *ERCC6* is involved in AMD. It is plausible that this involvement relates to oxidative stress and aging. Functional studies have implicated oxidative stress in the RPE in the aetiology of AMD [33], [34], while epidemiological studies unanimously consider aging as the main risk for AMD [35]. At the same time, *ERCC6* plays a role in *repair* of (oxidative) DNA damage. Loss of function mutations in *ERCC6* cause the autosomal recessive disorder Cockayne syndrome, which includes many severe physical and neurologic features, with premature aging and retinopathy as hallmarks of the disease. These observations led to the hypothesis that (oxidative) DNA damage and insufficient DNA repair contribute to aging pathology, including AMD [36]. This hypothesis was supported by a recent study, which compared DNA repair capacity in *leukocytes* of AMD patients and controls: Wozniak and co-workers found that AMD patients had higher levels of endogenous oxidative DNA damage and a lower repair capacity of DNA lesions [37].

We found that early AMD affected eyes had an almost 50% reduced expression of *ERCC6* in the RPE. In CS patients both *ERCC6* alleles carry loss of function mutations [20], [21]. To our knowledge, 50% reduction in transcription levels in CS carriers has not been reported to lead to pathology. Yet, it should be noted, that clinical data on CS carriers are scarce and, most likely, they have not been screened for retinal (AMD) pathology. Interestingly, repair of the 8-oxoguanine DNA lesion was investigated in a CS family: It was found that cells from the heterozygous parents had intermediate levels of repair of this oxidative DNA lesion compared to their CS affected and unaffected siblings [38]. It is important in this respect that most retinal cells, including the RPE are post-mitotic cells and that they are exposed to extremely high levels of oxidative stress over the prolonged period of (a life-) time. It is therefore conceivable that in this particular organ, the repair of oxidative DNA damage may be even more demanding than in other tissues. Small but prolonged shortcomings in DNA repair may, eventually, at advanced age become manifest in more damage and cell death than in other tissues. In this way, reduced levels of *ERCC6* in the RPE may well contribute to the development of AMD.

The *cause* of the reduced *ERCC6* transcription in RPE of early AMD affected eyes is not clear. However, given its implication in the multifactorial disorder AMD, and our inability to associate common *ERCC6* SNPs with AMD, we hypothesize that the genetic involvement of *ERCC6* in AMD, if any, may reside in (combination of) rare SNPs. Alternatively, the reduction in *ERCC6* transcription levels in relation to AMD may also be due to other genetic factors or to environmental insults, such as prolonged smoking.

### Conclusion

In summary, our study did not confirm a consistent relation between common genetic variation in *ERCC6* and AMD. We did not find a significant interaction between *ERCC6* SNP rs3793784 and either smoking, *CFH* or *LOC387715*. Using RPE from human donor eyes, we also could not confirm rs3793784 genotype dependent expression of *ERCC6*.

We did find significantly lower expression levels of *ERCC6* in human RPE affected by early AMD, regardless of the rs3793784 genotype. The latter finding suggests that *ERCC6* may be functionally implicated in the aetiology of AMD. Combining our data with those of the literature, we hypothesize that the AMD-related reduced transcriptional activity of *ERCC6* expression may be caused by diverse, small and heterogeneous genetic and/or environmental determinants.

## Materials and Methods

### Ethics Statement

All studies were approved by the Ethics Committees of the Academic Medical Center Amsterdam, the Erasmus Medical Center Rotterdam, the Institutional Review Board of Columbia University and the Age-Related Eye Disease Study (AREDS) Access Committee. All studies followed the tenets of the Declaration of Helsinki. All participants provided signed informed consent for participation in the study, retrieval of medical records, and use of blood and DNA for AMD research.

### Cases and controls

Altogether, we utilized one prospective, population-based study and three case-control studies consisting of 2682 AMD cases and 3152 ethnically- and age-matched control subjects.

### Study populations

Genetic association of the *ERCC6* SNP rs3793784 was analyzed in the Rotterdam Study and, in parallel, in the AMRO-NL case-control study population. Next, replication was carried out in two non-European study populations (Columbia University and AREDS). Finally, nine *ERCC6* (tag) SNPs were genotyped in the AMRO-NL study population.

*In The Rotterdam Study* all inhabitants of 55 years or older living in a suburb of Rotterdam, the Netherlands, were invited to participate [39], [40]. The initial cohort consisted of 10,275 eligible individuals, of whom 7,983 (78%) participated (98% Caucasian). The ophthalmologic part of the study consisted of 9,774 eligible individuals, of whom 7,598 (78%) participated. Baseline examinations took place from 1990 to 1993; three follow-up examinations were performed in 1993–1994, 1997–1999, and 2000–2005. Incident cases were defined as the absence of AMD in both eyes at baseline and its first appearance in at least 1 eye at follow-up.

*The AMRO-NL study population* consisted of 375 unrelated AMD patients and 199 controls. All subjects were Caucasian and recruited from the Netherlands Institute of Neuroscience Amsterdam and Erasmus University Medical Centre Rotterdam (Amsterdam-Rotterdam-NL (AMRO-NL)), through newsletters, via patient organizations, and nursing home visits. Controls were aged 65 years and older, and were unaffected spouses or non-related acquaintances of cases, or individuals who attended the ophthalmology department for reasons other than retinal pathology.

Cases and controls from *the Columbia University study population*, consisted of 368 unrelated individuals with AMD and 368 unrelated controls of European American descent, recruited at Columbia University (New York, NY) as previously described [6].

*The AREDS study population* was initially conceived as a long-term multicenter, prospective study of the clinical course of AMD and age-related cataract. Briefly, patients over the age of 55 years who self-reported to participating eye clinics between 1992 and 1996 were considered for AREDS, and eligibility was based on AMD symptoms and retinal photographs. A total of 1,699 patients were selected and enrolled in the clinical trial, all were put into one of five AMD severity categories, and then randomized into intervention and non-intervention groups. In this sample set, there were 929 AMD cases and 217 patients, free of AMD, acted as controls. The full details and results of the original AREDS study are reported elsewhere [41], [42].

### Diagnosis of AMD

Study subjects from the Rotterdam, AMRO-NL and Columbia University study populations underwent ophthalmic examination and fundus photography covering a 35° field centered on the macula after pupil dilatation at each visit (Topcon TRV-50VT fundus camera, Topcon Optical Co, Tokyo, Japan). Signs of AMD in those study populations were graded according to (a modification of) the international classification and grading system for AMD [43]. Cases and controls from the AREDS study were classified as having or not having disease on the basis of the AREDS classification system [44]. Although AREDS uses slightly different criteria for classification of early AMD and GA, the grading criteria were very similar for the four studies. Controls showed no, or less than five small hard drusen, and no other macular pathology. Early AMD cases had either soft distinct drusen with pigmentary irregularities or soft indistinct drusen with or without pigmentary irregularities. Late AMD cases presented with dry AMD, wet AMD, or a combination of both (mixed AMD).

### Genotyping

Standard DNA techniques were applied as described elsewhere [45]. Genotyping of the polymorphisms rs3793784 (*ERCC6 c.-6530C>G*), rs1061170 (*CFH Y402H*) and rs10490924 (*LOC387715 A69S*) were done with the Taqman assay (Applied Biosystems, Foster City, CA). Genotyping of the nine *ERCC6* (tag) SNPs was performed using an Illumina GoldenGate assay on a BeadStation 500 GX (Illumina Inc., San Diego,CA, US).

### ERCC6 Tag SNP selection for Illumina GoldenGate assay

Nine SNPs were selected to span and tag the entire *ERCC6* gene. SNP data were used from the Centre d'Étude du Polymorphisme Humain (CEPH) population (Utah residents with ancestry from northern and western Europe) by use of the International HapMAP Project. Available at: http://www.hapmap.org/, NCBI build 36, dbSNP b126; Accessed January 7, 2009. SNP selection was based on criteria like functional relevance, minor allele frequency (MAF)>10%, coverage of the main linkage disequilibrium (LD) blocks and tagging of the most common haplotypes. Tag SNPs were selected by use of Tagger, an option of Haploview [31] (all SNPs were captured with a LD tagging criteria of *r^2^*>0.8).

### Human donor eyes and ERCC6 expression

#### Postmortem eyes

Studies on human eye tissue were carried out in accordance with the Declaration of Helsinki on the use of human material for research. Donor eyes were obtained from the Corneabank Amsterdam. The method of selection of eyes for this study was essentially described elsewhere [18]. In summary, medical history of the donors revealed no pre-existing disorders, prolonged medication, or other prolonged agonal states that could possibly influence RPE gene expression or mRNA quality. Histological analysis was performed on all donor eyes, e.g. by staining with the periodic acid Schiff staining protocol (of sections (10 mm long) of the macula to identify and quantify drusen. Donor eyes were categorized into (1) “(old) healthy”, if the donor was older than 70 and histology revealed no drusen (on average 0–1 per 10 macular cryo-sections), and (2) “early AMD”, if the donor was older than 70 and histology showed 30 or more drusen per 10 macular cryo-sections with a still intact RPE.

#### Rs3793784 genotyping

Tuo et al. (2005) showed that *ERCC6* rs3793784, located in the untranslated 5′ flanking region of the gene, influenced *ERCC6* expression level: the G allele resulted in 2–3 times higher *ERCC6* expression than the C variant [19]. Therefore, genotyping of this SNP was performed on all above described donor eyes. Genomic DNA was prepared from frozen bulbi by standard extraction protocol using phenol. PCR and direct sequence analysis was carried out as previously described [46] with the use of the ABI-310 automated sequencer (Applied Biosystems).

#### *ERCC6* mRNA expression in the RPE of human donor eyes

We measured *ERCC6* mRNA expression in the RPE cells with real-time qPCR, in triplicate [47]. In summary, human donor eyes were snap-frozen in isopentane (Sigma-Aldrich, England) and stored at −80°C. Cryosections of 20 µm from the macula were cut and RPE cells were dissected using a PALM laser dissection microscope (P.A.L.M. Micro Laser Technologies AG). Total RNA was isolated with RNeasy mini (Qiagen) and amplified with the MessageAmp aRNA kit (Ambion). Template cDNA for the real-time PCR was made by reverse transcription of 200 ng aRNA with Superscript III (Invitrogen). Real-time PCR reactions were carried out in a 20 µl volume using qPCR Core Kit Sybr Green I (Eurogentec) and the following primer set: 5′-5′AAATCTGTGCACTTTCCATAGAACTTC-3′ and 5′- Reverse: TATTCTGGCTTGAGTTTCCAAATTC-3′. The levels of amplified product were detected by real-time monitoring of SYBR Green I dye fluorescence in the ABI Prism 7300 (Applied Biosystems). Expression levels of *ERCC6* were normalized using the geo-mean of the expression of internal control genes *RPLP0*, *PPIA*, and *EEF1a1* [47], [48]. Over ten different combinations of primersets were tested to obtain, in our hands and under our experimental conditions, the optimum reference gene set for genes expressed in the RPE (and/or photoreceptors). Two way ANOVA was used to determine the independent effect of (AMD) status and rs3793784 genotype on the mean expression as well as the interaction between these two variables. Significance was accepted at a *P* value of <0.05.

### Statistical analysis

Characteristics of the Dutch participants were compared among those affected and non-affected with analysis of covariance for continuous variables, and with logistic regression analysis for discrete variables adjusting for age and sex.

Hardy-Weinberg Equilibrium (HWE) of the *ERCC6*, *CFH* and *LOC387715* genotype distributions were tested using a Chi^2^ test.

In the Rotterdam Study, odds ratio's for prevalent AMD were estimated with logistic regression analyses, and relative risks for incident AMD were estimated with Cox proportional hazards analyses. Logistic regression was used in the AMRO-NL and the replication studies of Columbia University and AREDS to calculate odds ratios (ORs) and 95% confidence intervals (CI) in SPSS for windows (release 16.0; SPSS, Inc) for risk of AMD with the major alleles as reference. For the Rotterdam Study and the AMRO-NL study population, ORs were adjusted for age and sex.

Interaction with *ERCC6 c.-6530C>G* on AMD was determined for smoking, *CFH Y402H,* and *LOC387715 A69S* contrasting late AMD with no AMD. Analyses were initially performed on separate data sets of the Rotterdam Study (prevalent AMD, incident AMD), the AMRO-NL case-control study (in parallel), and subsequently conducted on combined data from the Netherlands (of population-based and case-control groups) and data of all population studies combined. Statistical significance for biological interaction was determined by calculating the synergy index (SI), which measures deviation from additivity of two risk factors [49], [50]. Two way ANOVA analysis was performed in SPSS for windows (release 16.0; SPSS, Inc).

## Supporting Information

Table S1 Baseline characteristics of the study populations.
(0.05 MB DOC)

Table S2 Risk of age-related macular degeneration for *ERCC6* c.-6530C>G genotypes. Abbreviations: AMD, age-related macular degeneration; OR, odds ratio; HR, hazard ratio The ORs and HRs are estimates of the relative risk of AMD, and represent the risk of disease (AMD vs stage 0) in the genetic risk group divided by the risk of disease (AMD vs stage 0) in the nonrisk group (noncarriers).a adjusted for sex, age.
(0.10 MB DOC)

Table S3 Odds Ratios (OR) and 95% Confidence Intervals (CI) of early and late age-related macular degeneration cases versus unrelated controls of the Amsterdam study population for single nucleotide polymorphisms in the *ERCC6* gene. AMD  =  age- related macular degeneration; MAF  =  minor allele frequency. “A” indicates common allele, “a” minor allele. Percentages not always 100% because of rounding. ORs are estimated with logistic regression analysis (with the control group as reference group and respectively early and late AMD as outcome variable). Adjusted for age and sex.
(0.10 MB DOC)

Table S4 RT-PCR analysis of *ERCC6* gene expression in RPE from eye donors with early AMD or non-affected age-matched donors. Both raw expression values for the three selected RPE housekeeping genes (RPLP0, PPIA, and EEF1a1) and ERCC6, as well as the resulting normalized expression values for ERCC6 are presented. AMD =  age- related macular degeneration. Normalization =  normalized to the geometric mean of three housekeeping genes (RPLP0, PPIA, and EEF1a1) [46], [47]. For statistical details see material and methods.
(0.06 MB DOC)
