# Supplementary material for: The ERCC6 Gene and Age-Related Macular Degeneration
Source: PLoS One. 2010 Nov 1;5(11):e13786. doi: 10.1371/journal.pone.0013786 (PMC2967476; doi:10.1371/journal.pone.0013786)
Supplement: Table S1 — Baseline characteristics of the study populations. (0.05 MB DOC) [file pone.0013786.s001.doc]

**Table S1: Baseline Characteristics of the Study Populations**

a. The Rotterdam Study

|  | ***ERCC6* c.-6530C>G**  **Noncarrier (N=1872)** | ***ERCC6* c.-6530C>G**  **Heterozygous (N=2790)** | ***ERCC6* c.-6530C>G**  **Homozygous (N=1062)** |
| --- | --- | --- | --- |
| Age, mean (sd), y |  |  |  |
| At baseline | 68.87 (8.60) | 68.63 (8.66) | 68.92 (8.70) |
| At diagnose | 73.05 (8.08) | 72.83 (8.22) | 73.10 (8.14) |
| Women, No (%) | 1071 (58.6) | 1620 (58.1) | 638 (60.1) |
| Smoking status, No/Total (%) |  |  |  |
| Never | 617/1793 (34.4) | 921/2757 (33.4) | 377/1048 (36.0) |
| Past | 751/1793 (41.9) | 1173/2757 (42.5) | 459/1048 (43.8) |
| Current | 425/1793 (23.7) | 663/2757 (24.0) | 212/1048 (20.2) |
| CFH Y402H |  |  |  |
| Noncarrier | 750/1789 (41.9) | 1130/2727 (41.4) | 416/1036 (40.2) |
| heterozygous | 802/1789 (44.8) | 1215/2727 (44.6) | 473/1036 (45.7) |
| homozygous | 237/1789 (13.2) | 382/2727 (14.0) | 147/1036 (14.2) |
| LOC387715 A69S |  |  |  |
| Noncarrier | 1147/1805 (63.5) | 1733/2771 (62.5) | 666/1058 (62.9) |
| heterozygous | 584/1805 (32.4) | 928/2771 (33.5) | 368/1058 (34.8) |
| homozygous | 74/1805 (4.1) | 110/2771 (4.0) | 24/1058 (2.3)* |

* *P*<.05 compared to ERCC6 c.-6530 C>G noncarrier

b. The AMRO-NL study population

|  | ***ERCC6* c.-6530C>G**  **Noncarrier (N=157)** | ***ERCC6* c.-6530C>G**  **Heterozygous (N=257)** | ***ERCC6* c.-6530C>G**  **Homozygous (N=87)** |
| --- | --- | --- | --- |
| Age, mean (sd), y | 76.22 (7.17) | 77.26 (7.20) | 76.71 (8.79) |
| Women, No (%) | 90 (57.3) | 150 (48.4) | 44 (50.6) |
| Smoking status, No/Total (%) |  |  |  |
| Never | 52/131 (39.7) | 68/219 (31.1) | 26/79 (32.9) |
| Past | 64/131 (48.9) | 105/219 (47.9) | 45/79 (57.0) |
| Current | 15/131 (11.5) | 46/219 (21.0)* | 8/79 (10.1) |
| *CFH Y402H*, No/Total (%) |  |  |  |
| Noncarrier | 37/150 (24.7) | 65/241 (27.0) | 21/81 (25.9) |
| Heterozygous | 74/150 (49.3) | 127/241 (52.7) | 39/81 (48.1) |
| Homozygous | 39/150 (26.0) | 49/241 (20.3) | 21/81 (25.9) |

* *P*<.05 compared to ERCC6 c.-6530 C>G noncarrier
