# Supplementary material for: The ERCC6 Gene and Age-Related Macular Degeneration
Source: PLoS One. 2010 Nov 1;5(11):e13786. doi: 10.1371/journal.pone.0013786 (PMC2967476; doi:10.1371/journal.pone.0013786)
Supplement: Table S2 — Risk of age-related macular degeneration for ERCC6 c.-6530C>G genotypes. Abbreviations: AMD, age-related macular degeneration; OR, odds ratio; HR, hazard ratio The ORs and HRs are estimates of the relative risk of AMD, and represent the risk of disease (AMD vs stage 0) in the genetic risk group divided by the risk of disease (AMD vs stage 0) in the nonrisk group (noncarriers).a adjusted for sex, age. (0.10 MB DOC) [file pone.0013786.s002.doc]

| **Table S2: Risk of Age-related Macular Degeneration for** *ERCC6* c.-6530C>G **Genotypes** | | | | | |  |  |  |  |  |  |
| --- | --- | --- | --- | --- | --- | --- | --- | --- | --- | --- | --- |
| 1. Based on prevalent cases | |  |  |  |  |  |  |  |  |  |  |
| a. The Rotterdam study | |  |  |  |  | Separate subtype analysis for the late AMD cases | | | | | |
|  | No AMD (controls) (n=3629) | Early AMD (n=427) | | Late AMD (n=78) | | GA (n=32) | | NMD (n=29) | | MIX (n=17) | |
|  | N (%) | N (%) | OR^a^ | N (%) | OR^a^ | N(%) | OR^a^ | N (%) | OR^a^ | N (%) | OR^a^ |
| *ERCC6* c.-6530C>G |  |  |  |  |  |  |  |  |  |  |  |
| Noncarrier | 1157 (31.9) | 138 (32.3) | 1 | 22 (28.2) | 1 | 10 (31.3) | 1 | 6 (20.7) | 1 | 6 (35.3) | 1 |
| heterozygous | 1795 (49.5) | 204 (47.8) | 0.99 (0.78-1.26) | 35 (44.9) | 1.01 (0.57-1.77) | 13 (40.6) | 0.82 (0.35-1.91) | 15 (51.7) | 1.62 (0.62-4.22) | 7 (41.2) | 0.65 (0.20-2.08) |
| homozygous | 677 (18.7) | 85 (19.9) | 1.12 (0.83-1.50) | 21 (26.9) | 1.71 (0.90-3.25) | 9 (28.1) | 1.51 (0.60-3.84) | 8 (27.6) | 2.31 (0.79-6.78) | 4 (23.5) | 1.14 (0.30-4.37) |
| MAF (%) | 0.43 | 0.44 |  | 0.49 |  | 0.48 |  | 0.53 |  | 0.44 |  |
|  |  |  |  |  |  |  |  |  |  |  |  |
| b. The AMRO-NL study population | |  |  |  |  | Separate subtype analysis for the late AMD cases | | | | | |
|  | No AMD (controls) (n=170) | Early AMD (n=84) | | Late AMD (n=247) | | GA (n=48) | | NMD (n=167) | | MIX (n=32) | |
|  | N (%) | N (%) | OR^a^ | N (%) | OR^a^ | N (%) | OR^a^ | N (%) | OR^a^ | N (%) | OR^a^ |
| *ERCC6* c.-6530C>G |  |  |  |  |  |  |  |  |  |  |  |
| Noncarrier | 53 (31.2) | 31 (36.9) | 1 | 73 (29.6) | 1 | 15 (31.3) | 1 | 48 (28.7) | 1 | 10 (31.3) | 1 |
| heterozygous | 92 (54.1) | 38 (45.2) | 0.60 (0.32-1.09) | 127 (51.4) | 0.90 (0.56-1.44) | 23 (47.9) | 0.83 (0.36-1.90) | 85 (50.9) | 0.87 (0.52-1.45) | 19 (59.4) | 0.80 (0.32-2.03) |
| homozygous | 25 (14.7) | 15 (17.9) | 0.93 (0.42-2.09) | 47 (19.0) | 1.37 (0.72-2.59) | 10 (20.8) | 1.33 (0.46-3.88) | 34 (20.4) | 1.47 (0.75-2.88) | 3 (9.4) | 0.42 (0.09-2.03) |
| MAF (%) | 0.42 | 0.41 |  | 0.45 |  | 0.45 |  | 0.46 |  | 0.39 |  |
|  |  |  |  |  |  |  |  |  |  |  |  |
| 2. Based on incident cases | |  |  |  |  |  |  |  |  |  |  |
| The Rotterdam study | |  |  |  |  | Separate subtype analysis for the late AMD cases | | | | | |
|  |  | Early AMD (n=509) | | Late AMD (n=93) | | GA (n=37) | | NMD (n=40) | | MIX (n=16) | |
|  |  | N (%) | HR^a^ | N (%) | HR^a^ | N (%) | HR^a^ | N (%) | HR^a^ | N (%) | HR^a^ |
| *ERCC6* c.-6530C>G |  |  |  |  |  |  |  |  |  |  |  |
| Noncarrier |  | 162 (31.8) | 1 | 21 (22.6) | 1 | 10 (27.0) | 1 | 8 (20.0) | 1 | 3 (18.8) | 1 |
| heterozygous |  | 257 (50.5) | 1.12 (0.92-1.37) | 50 (53.8) | **1.70(1.02-2.82)** | 21 (56.8) | 1.55 (0.73-3.29) | 22 (55.0) | 1.93 (0.86-4.33) | 7 (43.8) | 1.74 (0.45-6.72) |
| homozygous |  | 90 (17.7) | 0.99 (0.77-1.29) | 22 (23.7) | **1.92 (1.05-3.49)** | 6 (16.2) | 1.12 (0.41-3.09) | 10 (25.0) | 2.36 (0.93-6.00) | 6 (37.5) | 3.67 (0.92-14.69) |
| MAF (%) |  | 0.43 |  | 0.51 |  | 0.45 |  | 0.53 |  | 0.59 |  |
|  |  |  |  |  |  |  |  |  |  |  |  |
| 3. Based on prevalent and incident cases | |  |  |  |  |  |  |  |  |  |  |
| Pooled data from the Rotterdam study and the AMRO-NL study population | | | | |  | Separate subtype analysis for the late AMD cases | | | | | |
|  | No AMD (controls) (n=2567) | Early AMD (n=967) | | Late AMD (n=418) | | GA (n=115) | | NMD (n=229) | | MIX (n=74) | |
|  | N (%) | N (%) | OR^a^ | N (%) | OR^a^ | N (%) | ORa | N (%) | ORa | N (%) | ORa |
| *ERCC6* c.-6530C>G |  |  |  |  |  |  |  |  |  |  |  |
| Noncarrier | 840 (32.7) | 319 (33.0) | 1 | 116 (27.8) | 1 | 35 (30.4) | 1 | 61 (26.6) | 1 | 20 (27.0) | 1 |
| heterozygous | 1254 (48.9) | 470 (48.6) | 0.99 (0.84-1.17) | 212 (50.7) | 1.22 (0.95-1.57) | 55 (47.8) | 1.08 (0.69-1.68) | 119 (52.0) | 1.30 (0.94-1.80) | 38 (51.4) | 1.27 (0.73-2.22) |
| homozygous | 473 (18.4) | 178 (18.4) | 1.00 (0.80-1.24) | 90 (21.5) | **1.39 (1.02-1.89)** | 25 (21.7) | 1.27 (0.74-2.17) | 49 (21.4) | 1.43 (0.96-2.13) | 16 (21.6) | 1.45 (0.73-2.86) |
| MAF (%) | 0.43 | 0.43 |  | 0.47 |  | 0.46 |  | 0.47 |  | 0.47 |  |
| Abbreviations: AMD, age-related macular degeneration; OR, odds ratio; HR, hazard ratio  The ORs and HRs are estimates of the relative risk of AMD, and represent the risk of disease (AMD vs stage 0) in the genetic risk group divided by the risk of disease (AMD vs stage 0) in the nonrisk group (noncarriers).^a^ adjusted for sex, age | | | | | | | | | | | |
