# Supplementary material for: The ERCC6 Gene and Age-Related Macular Degeneration
Source: PLoS One. 2010 Nov 1;5(11):e13786. doi: 10.1371/journal.pone.0013786 (PMC2967476; doi:10.1371/journal.pone.0013786)
Supplement: Table S3 — Odds Ratios (OR) and 95% Confidence Intervals (CI) of early and late age-related macular degeneration cases versus unrelated controls of the Amsterdam study population for single nucleotide polymorphisms in the ERCC6 gene. AMD = age- related macular degeneration; MAF = minor allele frequency. “A” indicates common allele, “a” minor allele. Percentages not always 100% because of rounding. ORs are estimated with logistic regression analysis (with the control group as reference group and respectively early and late AMD as outcome variable). Adjusted for age and sex. (0.10 MB DOC) [file pone.0013786.s003.doc]

| **Table S3. Odds Ratios (OR) and 95% Confidence Intervals (CI) of Early and Late Age-related Macular Degeneration Cases Versus Unrelated Controls of the Amsterdam Study population for Single Nucleotide Polymorphisms in the ERCC6 Gene** | | | | | |  |
| --- | --- | --- | --- | --- | --- | --- |
|  | **No AMD (controls)** | **Early AMD** | | **Late AMD** | | |
| ***rs12220258*** | N=193 | N=93 |  | N=276 |  | |
|  | No. (%) | No. (%) | OR (95% CI) | No. (%) | OR (95% CI) | |
| Genotype |  |  |  |  |  | |
| Noncarrier (AA) | 132 (68.4) | 67 (72.0) | 1 | 198 (71.7) | 1 | |
| Heterozygous (Aa) | 55 (28.5) | 25 (26.9) | 0.92 (0.52-1.62) | 71 (25.7) | 0.87 (0.56-1.34) | |
| Homozygous (aa) | 6 (3.1) | 1 (1.1) | 0.35 (0.04-3.02) | 7 (2.5) | 0.87 (0.27-2.80) | |
| MAF (%) | 0.17 | 0.15 |  | 0.15 |  | |
| ***rs2228528*** | N=192 | N=93 |  | N=276 |  | |
|  | No. (%) | No. (%) | OR (95% CI) | No. (%) | OR (95% CI) | |
| Genotype |  |  |  |  |  | |
| Noncarrier (AA) | 132 (68.8) | 67 (72.0) | 1 | 198 (71.7) | 1 | |
| Heterozygous (Aa) | 54 (28.1) | 25 (26.9) | 0.93 (0.53-1.65) | 70 (25.4) | 0.86 (0.56-1.33) | |
| Homozygous (aa) | 6 (3.1) | 1 (1.1) | 0.35 (0.04-3.02) | 7 (2.5) | 0.86 (0.27-2.78) | |
| MAF (%) | 0.17 | 0.15 |  | 0.15 |  | |
| ***rs2228529*** | N=188 | N=93 |  | N=275 |  | |
|  | No. (%) | No. (%) | OR (95% CI) | No. (%) | OR (95% CI) | |
| Genotype |  |  |  |  |  | |
| Noncarrier (AA) | 117 (62.2) | 54 (58.1) | 1 | 183 (66.5) | 1 | |
| Heterozygous (Aa) | 62 (33.0) | 33 (35.5) | 1.19 (0.69-2.05) | 81 (29.5) | 1.19 (0.69-2.05) | |
| Homozygous (aa) | 9 (4.8) | 6 (6.5) | 1.75 (0.58-5.32) | 11 (4.0) | 1.75 (0.58-5.32) | |
| MAF (%) | 0.21 | 0.24 |  | 0.19 |  | |
| ***rs2229760*** | N=192 | N=93 |  | N=272 |  | |
|  | No. (%) | No. (%) | OR (95% CI) | No. (%) | OR (95% CI) | |
| Genotype |  |  |  |  |  | |
| Noncarrier (AA) | 61 (31.8) | 30 (32.3) | 1 | 81 (29.8) | 1 | |
| Heterozygous (Aa) | 99 (51.6) | 48 (51.6) | 0.92 (0.52-1.62) | 142 (52.2) | 1.01 (0.65-1.57) | |
| Homozygous (aa) | 32 (16.7) | 15 (16.1) | 0.87 (0.41-1.88) | 49 (18.0) | 1.11 (0.62-1.99) | |
| MAF (%) | 0.42 | 0.42 |  | 0.44 |  | |
| ***rs2281793*** | N=193 | N=92 |  | N=275 |  | |
|  | No. (%) | No. (%) | OR (95% CI) | No. (%) | OR (95% CI) | |
| Genotype |  |  |  |  |  | |
| Noncarrier (AA) | 57 (29.5) | 26 (28.3) | 1 | 76 (27.6) | 1 | |
| Heterozygous (Aa) | 99 (51.3) | 49 (53.3) | 1.03 (0.58-1.86) | 139 (50.5) | 0.96 (0.62-1.50) | |
| Homozygous (aa) | 37 (19.2) | 17 (18.5) | 0.92 (0.44-1.95) | 60 (21.8) | 1.11 (0.63-1.94) | |
| MAF (%) | 0.45 | 0.45 |  | 0.47 |  | |
| ***rs4253165*** | N=194 | N=93 |  | N=277 |  | |
|  | No. (%) | No. (%) | OR (95% CI) | No. (%) | OR (95% CI) | |
| Genotype |  |  |  |  |  | |
| Noncarrier (AA) | 121 (62.4) | 54 (58.1) | 1 | 184 (66.4) | 1 | |
| Heterozygous (Aa) | 63 (32.5) | 33 (35.5) | 1.21 (0.71-2.08) | 82 (29.6) | 0.98 (0.64-1.49) | |
| Homozygous (aa) | 10 (5.2) | 6 (6.5) | 1.56 (0.53-4.64) | 11 (4.0) | 0.76 (0.30-1.90) | |
| MAF (%) | 0.21 | 0.24 |  | 0.19 |  | |
| ***rs4253211*** | N=193 | N=93 |  | N=277 |  | |
|  | No. (%) | No. (%) | OR (95% CI) | No. (%) | OR (95% CI) | |
| Genotype |  |  |  |  |  | |
| Noncarrier (AA) | 157 (81.3) | 79 (84.9) | 1 | 224 (80.9) | 1 | |
| Heterozygous (Aa) | 32 (16.6) | 14 (15.1) | 0.92 (0.46-1.84) | 49 (17.7) | 1.11 (0.67-1.83) | |
| Homozygous (aa) | 4 (2.1) | 0 (0.0) | sample size too small | 4 (1.4) | 0.89 (0.20-3.92) | |
| MAF (%) | 0.1 | 0.08 |  | 0.1 |  | |
| ***rs4253231*** | N=190 | N=93 |  | N=274 |  | |
|  | No. (%) | No. (%) | OR (95% CI) | No. (%) | OR (95% CI) | |
| Genotype |  |  |  |  |  | |
| Noncarrier (AA) | 159 (83.7) | 80 (86.0) | 1 | 226 (82.5) | 1 | |
| Heterozygous (Aa) | 29 (15.3) | 10 (10.8) | 0.71 (0.33-1.54) | 47 (17.2) | 1.08 (0.64-1.84) | |
| Homozygous (aa) | 2 (1.1) | 3 (3.2) | 3.09 (0.49-19.31) | 1 (0.4) | 0.30 (0.02-3.86) | |
| MAF (%) | 0.09 | 0.09 |  | 0.09 |  | |
| ***rs4838523*** | N=193 | N=91 |  | N=274 |  | |
|  | No. (%) | No. (%) | OR (95% CI) | No. (%) | OR (95% CI) | |
| Genotype |  |  |  |  |  | |
| Noncarrier (AA) | 155 (80.3) | 73 (80.2) | 1 | 213 (77.7) | 1 | |
| Heterozygous (Aa) | 38 (19.7) | 17 (18.7) | 0.89 (0.47-1.69) | 57 (20.8) | 1.05 (0.65-1.69) | |
| Homozygous (aa) | 0 (0.0) | 1 (1.1) | sample size too small | 4 (1.5) | sample size too small | |
| MAF (%) | 0.1 | 0.1 |  | 0.12 |  | |
| AMD = age- related macular degeneration; MAF = minor allele frequency. "A" indicates common allele, "a" minor allele. Percentages not always 100% because of rounding. ORs are estimated with logistic regression analysis (with the control group as reference group and respectively early and late AMD as outcome variable). Adjusted for age and sex. | | | | | | |
|
|
|
