# Supplementary material for: The ERCC6 Gene and Age-Related Macular Degeneration
Source: PLoS One. 2010 Nov 1;5(11):e13786. doi: 10.1371/journal.pone.0013786 (PMC2967476; doi:10.1371/journal.pone.0013786)
Supplement: Table S4 — RT-PCR analysis of ERCC6 gene expression in RPE from eye donors with early AMD or non-affected age-matched donors. Both raw expression values for the three selected RPE housekeeping genes (RPLP0, PPIA, and EEF1a1) and ERCC6, as well as the resulting normalized expression values for ERCC6 are presented. AMD = age- related macular degeneration. Normalization = normalized to the geometric mean of three housekeeping genes (RPLP0, PPIA, and EEF1a1) [46], [47]. For statistical details see material and methods. (0.06 MB DOC) [file pone.0013786.s004.doc]

***Table S4. RT-PCR analysis of ERCC6 gene expression in RPE from eye donors with early AMD or non-affected age-matched donors.***

|  | ***RAW VALUES*** | | | | ***NORMALIZED VALUES*** |
| --- | --- | --- | --- | --- | --- |
|  | ***RPLP0*** | ***PPIA*** | ***EEF1a1*** | ***ERCC6*** | ***ERCC6*** |
| ***Early AMD RPE donor eye nr*** |  |  |  |  |  |
| 1 | 127141.98 | 29794.97 | 266778.14 | 7.86 | **3.59** |
| 2 | 271949.82 | 27580.88 | 192751.28 | 21.18 | **8.58** |
| 3 | 39560.72 | 15669.60 | 58290.81 | 13.11 | **18.17** |
| 4 | 43006.91 | 7820.69 | 90704.15 | 7.31 | **10.72** |
| 5 | 37111.56 | 13161.91 | 67649.00 | 6.40 | **9.14** |
| 6 | 55537.74 | 12115.62 | 59859.02 | 6.05 | **8.09** |
| 7 | 206256.63 | 30139.77 | 126710.89 | 16.03 | **13.81** |
| 8 | 112524.60 | 8799.35 | 63831.50 | 7.30 | **8.40** |
| 9 | 32683.64 | 40352.79 | 108173.00 | 37.78 | **33.14** |
| 10 | 53523.36 | 16339.55 | 27788.13 | 8.05 | **7.96** |
| 11 | 37891.35 | 25164.13 | 105822.06 | 10.07 | **12.73** |
| 12 | 14801.76 | 4733.79 | 34014.94 | 4.81 | **9.91** |
| 13 | 317584.49 | 137167.67 | 441477.67 | 51.08 | **16.51** |
| 14 | 179980.48 | 61265.23 | 314561.14 | 45.61 | **8.74** |
| *Average* | *109254.00* | *30722.00* | *139887.00* | *17.33* | ***12.11*** |
| ***Matched old-healthy donor eye nr*** |  |  |  |  |  |
| 1 | 43492.56 | 53352.97 | 168767.89 | 47.12 | **29.51** |
| 2 | 223618.64 | 2641.75 | 264641.71 | 25.29 | **21.51** |
| 3 | 386653.29 | 208455.65 | 614043.33 | 218.49 | **27.27** |
| 4 | 104698.48 | 13235.10 | 141945.83 | 29.70 | **23.40** |
| 5 | 48304.87 | 4874.64 | 27375.18 | 4.28 | **10.55** |
| 6 | 6938.85 | 2173.92 | 15751.27 | 3.97 | **29.34** |
| 7 | 11254.24 | 7131.95 | 40504.12 | 6.34 | **19.61** |
| 8 | 14144.77 | 1673.07 | 18509.53 | 3.44 | **20.77** |
| 9 | 64436.07 | 9024.81 | 87293.16 | 12.64 | **15.64** |
| *Average* | *100394.00* | *33618.00* | *153204.00* | *39.03* | ***21.96*** |
|  |  |  |  |  |  |
| **p-value early AMD vs. old-healthy** |  | | | | ***0.018*** |

Both raw expression values for the three selected RPE housekeeping genes (*RPLP0*, *PPIA*, and *EEF1a1*) and *ERCC6, as well as* the resulting normalized expression values for *ERCC6 are* presented. AMD= age- related macular degeneration. Normalization= normalized to the geometric mean of three housekeeping genes (*RPLP0*, *PPIA*, and *EEF1a1*) [46,47]. For statistical details see material and methods.
